# Establishment of indirect ELISA method for Salmonella antibody detection from ducks based on PagN protein

**DOI:** 10.1186/s12917-022-03519-7

**Published:** 2022-12-05

**Authors:** Shaopeng Hou, Shuyang Wang, Xinyuan Zhao, Wei Li, Jing Gao, Yanjun Wang, Ruihua Zhang, Lingling Gong, Shijin Jiang, Yanli Zhu

**Affiliations:** 1 Shandong Provincial Key Laboratory of Animal Biotechnology and Disease Control and Prevention, Shandong Provincial Engineering Technology Research Center of Animal Disease Control and Prevention, Department of Preventive Veterinary Medicine, College of Veterinary Medicine, Shandong Agricultural University, Taian, 271018 China; 2 Shandong Provincial Quality Inspection Center of Animal Feed and Veterinary Medicine, Jinan, 250010 China

**Keywords:** Duck-derived *Salmonella*, PagN protein, Prokaryotic expression, iELISA

## Abstract

**Background:**

*Salmonella* as an important food-borne zoonotic bacterial pathogen, infection in ducks is a recessive infection, however, it can also cause high mortality and threat to food safety. Preventing and controlling the infection and transmission of *Salmonella* in ducks critically require rapid and sensitive detection method. Full-length *Salmonella*-specific protein PagN was induced and expressed in *E.coil* BL21 and was purified as an antigen to establish an indirect enzyme-linked immunosorbent assays (iELSA) detection kit.

**Results:**

The recombinant PagN protein has a molecular weight of 43 kDa containing a His-tag, was recognized by an anti-*Salmonella* positive serum by Western blot assay. The optimal concentration of PagN as a coating antigen in the iELISA was 1 μg/mL, and the optimal dilution of enzyme-labeled secondary antibody was 1:4000 (0.025 μg/mL). The cutoff OD_450_ value was established at 0.268. The iELISA kit showed high selectivity since no cross-reaction with *E. coli, Staphylococcus aureus* and *Streptococcus* was observed. iELISA method and Dot-blot test were performed on 100 clinical sera samples collected from duck farms, and the actual coincidence rate was 89% (89/100). 613 duck serum samples from 3 different farms were tested using established method and commercial ELISA kit. The concordance between the two methods was 94.1%.

**Conclusion:**

Anti-PagN based iELISA can serve as a useful tool for diagnosis of *Salmonella* infection.

**Supplementary Information:**

The online version contains supplementary material available at 10.1186/s12917-022-03519-7.

## Background

Salmonellosis in ducks is a common and multiple infectious disease caused by different *Salmonella* serotypes, which seriously endangers the survival of ducklings. In particular, *Salmonella* co-infected with *Riemerella anatipestifer* and *E.coli* cause enormous morbidity and mortality [1]. *Salmonella* can be transmitted both horizontally and vertically [2], and lead to a large outbreak of *Salmonella* infection in a short time if it is not detected in time. In recent years, more and more multi-drug-resistant bacteria have appeared in China, which makes salmonellosis in ducks more difficult to prevent and control [3, 4]. At the same time, *Salmonella* is one of the most important foodborne pathogens worldwide. Duck meat and other by-products contaminated by *Salmonella* remains a potential source of human salmonellosis [5, 6]. *Salmonella* is 1 of the 4 key global causes of diarrhoeal diseases according to the World Health Organization (WHO) guidelines [7]. In the United States, around 20% of foodborne illnesses attributed to *Salmonella* can be associated with poultry and poultry products [8]. Moreover, the proportion is likely to be higher in developing countries. As the country with the largest duck-raising industry in the world, China needs to strengthen the detection and control of *Salmonella*. Therefore, the establishment of accurate and rapid diagnostic method is an important way to solve this problem.

Although detection methods relying on traditional bacterial culturing have been used as the gold standard for the detection of *Salmonella* [9], it is a time-consuming and laborious process, and cannot meet the urgent need of real-time detection. Some *Salmonella* detection methods have been developed such as: polymerase chain reaction (PCR) [10], quantitative real-time PCR (qPCR) [11, 12], loop-mediated isothermal amplification (LAMP) [13, 14], gold immunochromatographic test strip (ICTS) [15], enzyme-linked immunosorbent assays [16, 17], and biosensors [18, 19]. Among the above methods, conventional iELISA established based on the excellent antigenic protein relevant to high conservation from different serotypes and antigenic specificity is very suitable for rapidly monitoring and purifying the *Salmonella* contamination in large-scale duck industry in real time.

PagN is an outer membrane protein that contributes to adhesion to and invasion of epithelial cells by *Salmonella*. The *PagN* as conserved gene is localized on the specific centisome 7 genomic island and widely distributed among the different *Salmonella* serovars [20, 21]. The *PagN* is a PhoP-regulated gene that is up-regulated during growth within macrophages and in vivo in murine models of infection [22]. Some studies have shown that the PagN protein utilizes heparinated proteoglycans to adhere and invade mammalian cells [21, 23]. The PagN protein contains a transmembrane region and four extracellular loops. The latter is very important to the invasion of epithelial cells and can induce a strong immune response [20, 24]. After bioinformatics analysis, we found that there are many potential antigen sites for PagN protein, and the immunogenicity, hydrophilicity, flexibility and surface possibility are relatively good as antigenic determinants. Therefore, PagN could be a promising target for detecting *Salmonella* antibodies.

In this study, the *PagN* gene was cloned into the prokaryotic expression vector pET-32a(+) to construct a recombinant expression plasmid and expressed in *E. coli* BL 21 cells. An iELISA was developed based on purified recombinant PagN for detecting *Salmonella* antibodies in duck serum. This method has the potential to contribute to effective diagnosis of Salmonellosis in ducks and to gradually eliminate *Salmonella* infections.

## Results

### Serum preparation

We found that different positive sera and corresponding bacteria produced agglutination reaction. The liquid in the test tube was clear macroscopically, but the clumps were visible to the naked eye when a hanging drop was examined. More precipitate collected in the bottom of the tubes. On the contrary, there was no agglutination in each negative control group, and the liquid was turbid. The results showed that all the positive and negative sera were obtained, which can be used for the negative control, and sensitivity and specificity detection of iELISA.

### Screening of target protein

The homology of *PagN* gene among *Salmonella* reached more than 98%, and the homology with other non-*Salmonella* was less than 50% by BLASTN comparison (Fig. S1). DNAstar software and IEDB website predicted that the PagN protein (43 kDa) had a relatively large proportion of *β*-turn and random coil, and contained good hydrophilicity, flexibility, immunogenicity and surface possibility (Fig. S2). B-cell epitope prediction showed that four antigenic epitopes with strong antigenicity nearly distributed in the extracellular loop region of the protein, which could stimulate the body to produce specific antibodies for serological detection (Fig. S3).

### Recombinant plasmid construction and the recombinant PagN protein with his-tag purification

**Fig. 1:**
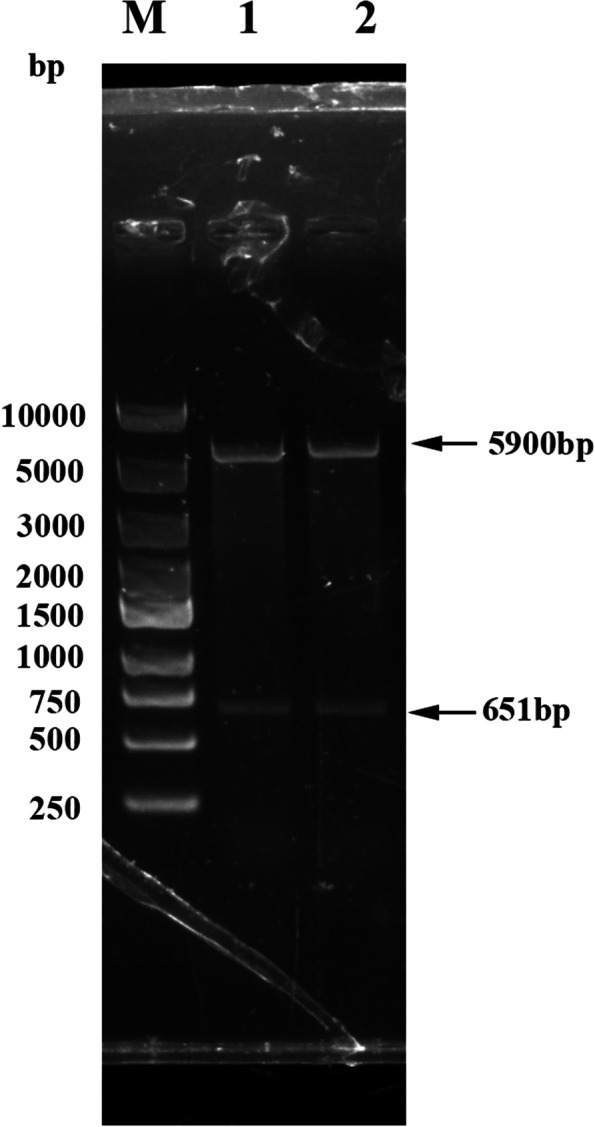
Agarose gel electrophoresis of the recombinant plasmid pET32-a(+)-PagN. M: DNA molecular weight marker; lane 1,2: *Bam*HI and *Hind*III double-digested product

**Fig. 2:**
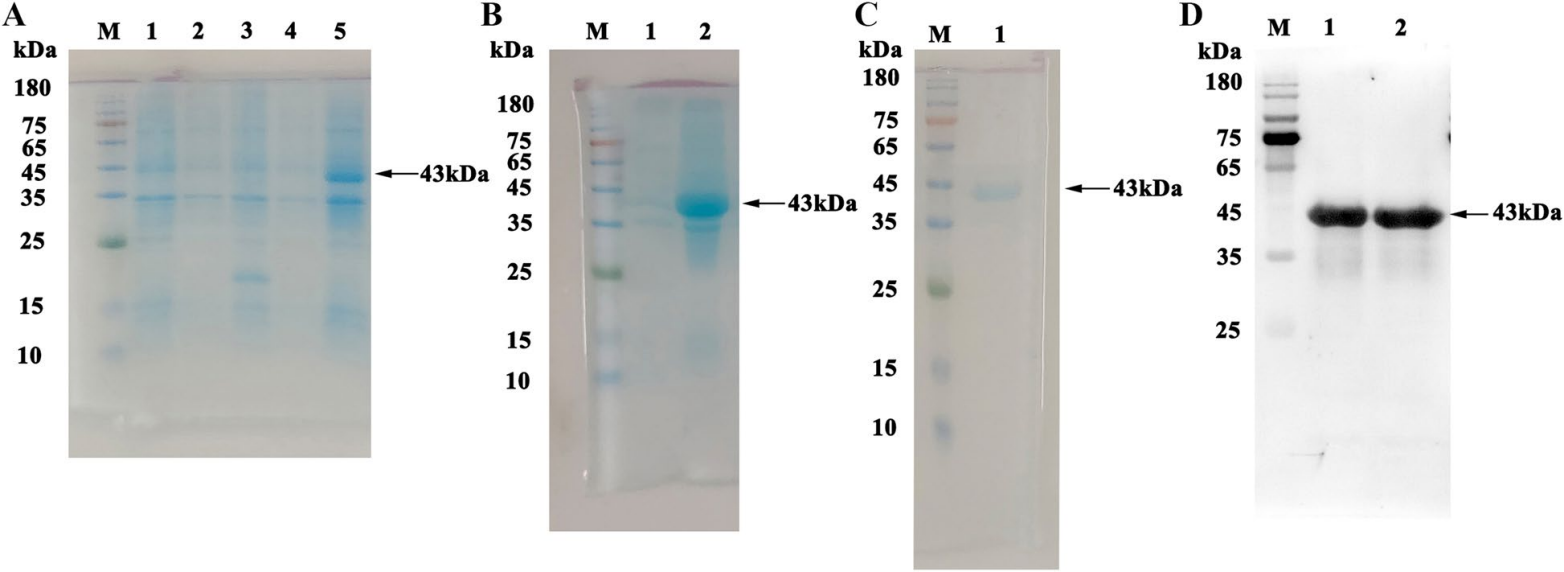
Recombinant PagN protein expression, purification and identification. (A) Induction of expression of the PagN protein. M: Protein molecular weight marker; Lane 1: *E.coil* BL21(DE3) cells; Lane 2: empty vector pET-32a(+) plasmid transferred into *E.coil* BL21(DE3) was induced expression without IPTG; Lane 3: empty vector pET-32a(+) plasmid transferred into *E.coil* BL21(DE3) was induced expression by IPTG; Lane 4: PagN recombinant protein expression without IPTG induction; Lane 5: PagN recombinant protein expression by IPTG induction at 37 °C for 6 h. (B) Analysis of recombinant PagN protein expression patterns. M: Protein molecular weight marker; Lane 1: expression of the target protein in supernatant; Lane 2: expression of the target protein in inclusion bodies. (C) Analysis of the purified recombinant PagN protein by affinity chromatography. M: Protein molecular weight marker; Lane 1: purified PagN protein using nickel affinity chromatography. (D) Western blotting analysis of recombinant PagN protein. M: Protein molecular weight marker; Lane 1, 2: recombinant PagN protein (43 kDa) identified via Western blotting

A 651 bp of the *PagN* gene sequence was successfully amplified by PCR from the *Salmonella* enteritis strain (CVCC 3377), which was subsequently inserted into the pET-32a(+) vector. The recombinant pET-32a (+)-PagN plasmid was identified by DNA sequencing and double digestion with BamH I and Hind III (Fig. 1). The plasmid was transformed into *E.coli* BL21 (DE3) cells. The recombinant PagN protein with N-terminal His-tag was induced for 6 h at 37 °C by adding 1 mM isopropyl-b-D-thiogalactopyranoside (IPTG). Sodium dodecyl sulfate polyacrylamide gel electrophoresis (SDS-PAGE) analysis showed that a target band was found at about 43 kDa (lane 5 in Fig. 2A), but no band could be detected for empty vector (lane 3 in Fig. 2A), indicating that recombinant PagN protein was successfully induced. Supernatant and inclusion body samples obtained after ultrasonication in an ice bath were processed, and then subjected to SDS-PAGE analysis showed that the recombinant PagN protein mainly expressed in the form of inclusion bodies (Fig. 2B). Ni^2+^ NTA agarose gel affinity chromatography was applied to purify the His-tagged recombinant PagN protein, and a single band was detected by SDS-PAGE (Fig. 2C). For western-blot analysis, the recombinant protein can be recognized by duck anti-*Salmonella* serum (Fig. 2D).

### Establishment of the recombinant PagN protein-based iELISA

The optimal coating antigen concentration as determined by checkerboard titration was 1 μg/mL, and the optimal serum dilution was identified as 1:50 (Table 1). The subsequent reaction conditions as following: the concentration of coating antigen was for 2 h at 37 °C; 5% skimmed milk powder blocked for 2 h at 37 °C; the best serum dilution was 1:50, the incubation condition of serum was at 37 °C for 30 min; the enzyme-labeled secondary antibody at a working dilution of 1:4000 was incubated 90 min at 37 °C, and the catalytic reaction time of tetramethylbenzidine (TMB) was 20 min (Table 2).

**Table 1:** Determination of optimal antigen coating concentration and serum dilution by chessboard method

Antigen coating concentration (μg/ml)		Serum dilution					
		1:25	1:50	1:100	1:200	1:400	1:800
0.25	+	1.615	1.152	0.881	0.625	0.403	0.270
	-	0.153	0.092	0.074	0.062	0.048	0.046
	P/N	10.558	12.473	11.959	10.027	8.389	5.877
0.5	+	1.888	1.354	0.999	0.726	0.514	0.336
	-	0.163	0.101	0.080	0.068	0.049	0.042
	P/N	11.559	13.406	12.440	10.620	10.426	8.008
1	+	2.051	1.435	1.055	0.760	0.515	0.334
	-	0.167	0.099	0.080	0.065	0.052	0.048
	P/N	12.279	**14.495**	13.137	11.628	9.910	6.965
2	+	2.160	1.495	1.077	0.762	0.518	0.348
	-	0.161	0.108	0.085	0.065	0.052	0.048
	P/N	13.416	13.882	12.671	11.784	9.904	7.207
4	+	2.383	1.861	1.301	0.931	0.609	0.370
	-	0.165	0.134	0.103	0.104	0.056	0.051
	P/N	14.413	13.925	12.594	8.920	10.805	7.296

**Table 2:** iELISA reaction procedure

Step	Dilution	Vol. (μL)/Well	Inc. period (min)	Temp. (°C)	Wsah × time (min)
Coating	Antigen 1 μg/mL	100	120	37	3×5
Blocking	5% skim milk	200	120	37	3×5
Adding serum	1:50 dilution	100	30	37	3×5
Adding second antibody	1:4000 dilution	100	90	37	3×5
Substrate	NA	100	20	37	NA
Stop	NA	50	Within 30 min	37	NA

### Cut-off value determination of the iELISA

**Fig. 3:**
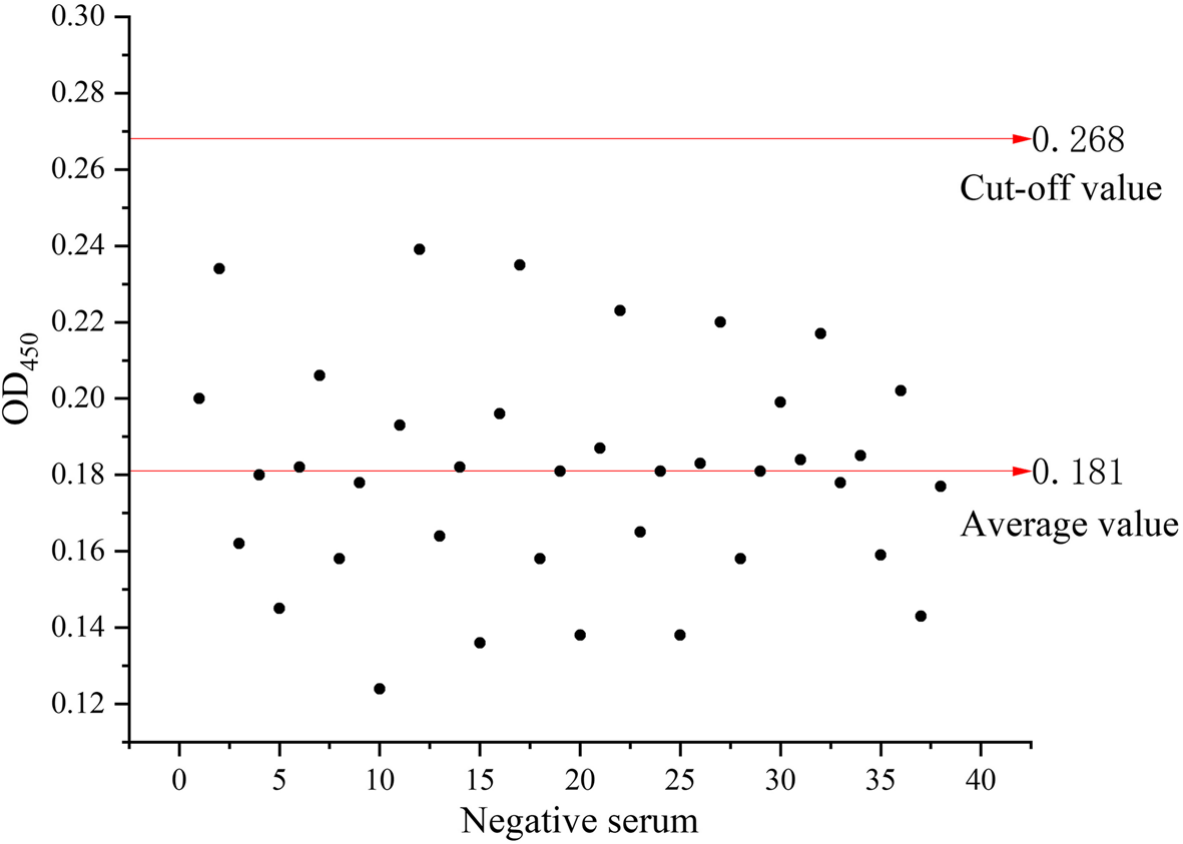
Determination of the cut-off value in iELISA method

The OD_450_ values of 38 *Salmonella* negative sera were determined by the established iELISA method. According to the cut-off value = mean value of negative sera + 3 × standard deviation, the cutoff value was determined to be 0.268 (Fig. 3).

### Sensitivity of the iELISA

**Fig. 4:**
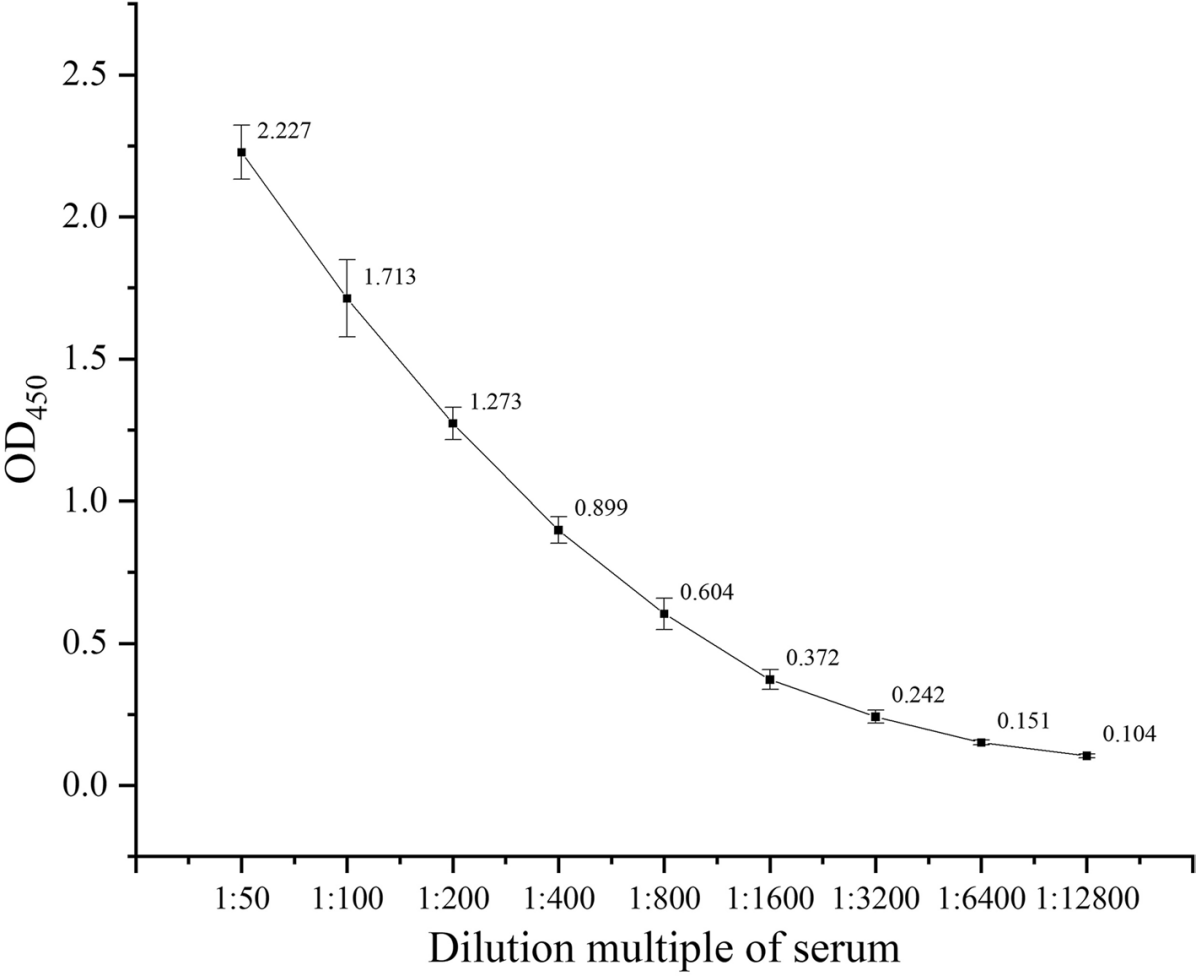
Sensitivity analysis of the iELISA

The *Salmonella* positive sera were continuously diluted by two-fold (1,50 to 112,800) and detected by iELISA. The maximum dilution was 1:1600 according to the established iELISA method (Fig. 4).

### Specific determination of the iELISA

**Fig. 5:**
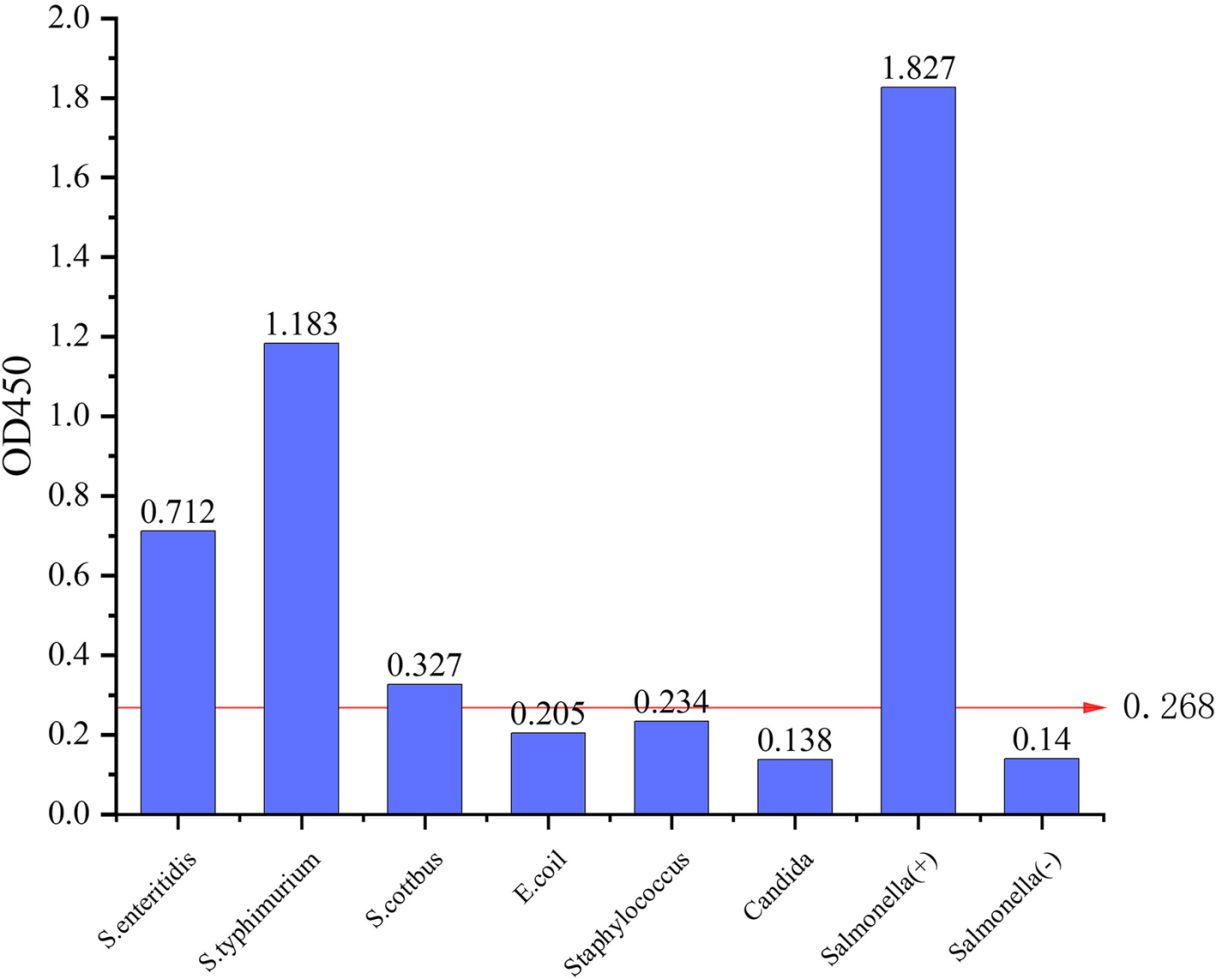
Specificity analysis of the iELISA

The serum cross-reactivity test results showed that the OD_450_ values of all 3 *Salmonella* serovar sera were higher than the cut-off value, which indicated that established iELISA method could detect the *Salmonella* infection in ducks. The positive serotest results for the remaining common bacteria were all negative, indicating that the iELISA is specific for duck-derived *Salmonella* (Fig. 5).

### Repeatability of the iELISA

Repeatability test results showed intragroup and intergroup coefficient of variation (CVs) less than 5.6 and 9.7%, respectively, indicating that the established iELISA method has good repeatability (Table 3).

**Table 3:** Determination of coefficient of variation (CV) from 4 serum samples

Samples	Intra-batch repetitive test				Inter-batch repetitive test		
	Average	SD	CV(%)		Average	SD	CV(%)
P1	2.285	0.076	3.3		1.490	0.112	7.5
P2	0.699	0.039	5.6		0.838	0.029	3.8
P3	1.019	0.032	3.1		0.873	0.084	9.7
N	0.160	0.007	4.1		0.115	0.008	7.2

P1,P2,P3: 3 positive serum samples; N:negative serum

### Comparison of iELISA with dot-blot

A total of 100 serum samples were used to evaluate the potential of developed ELISA by comparison with Dot-blot. The developed iELISA showed 89% coincidence rate with the Dot-blot (Table 4).

**Table 4:** Comparison of the iELISA and dot blot test

	Dot-Blot positive samples	Dot-Blot negative samples	Total
iELISA positive samples	52	4	56
iELISA negative samples	7	37	44
Coincidence rate (%)	89% (52/59)	90% (37/41)	89% (89/100)

### Application of iELISA method to clinical samples

A total of 613 serum samples were collected from 3 different duck farms. All serum samples were monitored using the iELISA and commercial ELISA kit (Table 5). The results showed that 257 and 320 serum samples tested positive and negative, respectively. The analysis of discrepant data was obtained for 26 sera that was positive by iELISA but be negative by commercial ELISA kit. 10 sera were negative by iELISA but positive by commercial kit. Therefore, there were good agreement (94.1%) between iELISA and commercial kit.

**Table 5:** Comparison of iELISA and commercial ELISA kit

	Commerical kit positive samples	Commerical kit negative samples	Total
iELISA positive samples	257	26	283
iELISA negative samples	10	320	330
Coincidence rate (%)	96.3% (257/267)	92.5% (320/346)	94.1% (577/613)

## Discussion

Duck industry is an important part of animal husbandry in China, and the quality of duck products is closely related to human health. Recent study has shown that 604 isolates of *Salmonella* from 3340 retail meat samples from 2009 to 2016, of which 241 strains were isolated from poultry (ducks and chickens), accounting for 40% of the total isolates [25]. These showed that *Salmonella* infection in poultry has been very serious. Compared with chickens, little attention has been paid to ducks Salmonellosis. Therefore, there is an urgent need to determine the prevalence of *Salmonella* in ducks, which help curb the spread of this pathogen in duck farms.

So far, more than 2600 identified serotypes display different pathogenicities and an extensive host range [26]. Many studies have reported that ducks can be infected with multiple serotypes of *Salmonella* [27, 28], therefore the established method needs to be capable of broadly detecting various serotypes. Since there is no immunization policy for duck salmonellosis in China, we use antibody testing to confirm whether ducks are infected with *Salmonella*. iELISA is a simple, high throughput and strong specificity method that can be used for rapid pathogen detection, and is very suitable for the current domestic large-scale duck farms. Some studies have used lysed bacterial cells or LPS as the detection antigen to establish an iELISA kit. Although this method can detect many serotypes of *Salmonella*, it is easy to lead to false positive detection results because of antigenic epitopes similar to those on other bacteria, such as *E. coli*. The method developed by lipopolysaccharide (LPS) limited specificity of the antigen-antibody interaction. To solve this problem, we screened a special PagN protein, it is an outer membrane protein that contribute to the virulence, and is widely distributed and well conserved among the different species and subspecies of *Salmonella*. There are both B cell and T cell antigen peptides in its four extracellular rings, which can induce a strong immune response. In recent years, it has often been studied as a candidate protein for subunit vaccines [21, 29, 30].

In this study, *E. coli* prokaryotic expression system was used to express the recombinant protein in vitro. Although the expression system do not modify the protein, is easy to form inclusion body protein. Compared with other protein expression systems, the genetic background of *E. coli* expression system is clear, the operation process is relatively simple, easy to purify, and the protein expression production is high. Therefore, the expression system can effectively reduce the cost of the kit [31].

In this study, the complete DNA sequence of *PagN* gene was obtained by PCR, and cloned into pET-32a(+) to construct successfully the recombinant prokaryotic expression plasmid pET-32a(+)-PagN. There is a 6 × His tag protein in the pET-32a (+) plasmid itself, and the tag is fused and expressed at both ends of the PagN protein [32]. So the target protein can be efficiently purified by nickel ion affinity chromatography. SDS-PAGE analysis showed that the target protein with high purity was obtained in this study, which effectively eliminated the interference of *E. coli* bacterial protein, and further improved the specificity of the iELISA method established. Because the protein was in the form of inclusion body, 8 M urea was used to denature the protein in the purification process, resulting in no biological activity of the protein. The dialysis method with appropriate concentration gradient is very efficient for protein refolding and has almost no effect on protein concentration [33]. We used this method to refold the protein and finally obtained the soluble active protein.

We initially established the iELISA assay for detecting *Salmonella* antibodies based on recombinant PagN protein, and determined the optimal reaction conditions. In the present study, we obtained a fixed cut-off of 0.268 for iELISA performance by detecting 38 *Salmonella* negative serum samples. The method has high specificity sensitivity when treated with *S. enteritidis*, *S. typhimurium* and *S. Kottbus* positive sera from ducks. At the same time, iEILSA method had no cross-reaction with antibodies against *E. coli*, *Staphylococcus aureus* and *Streptococcus*. Compared with the iELISA method established using PagC protein [34], the method we established is more accurate for *E. coli* positive serum, indicating that the method established in this study has better specificity. Although the sensitivity of this study was lower than previous studies, the minimum detection limit of the referenced positive sera was still 1:1600, which was also applicable to sera with lower antibody titers. The specificity of the test may be higher when the sensitivity is low, and the specific relationship between sensitivity and specificity in this study needs to be supported by more subsequent experimental data. Because of the strong subjectivity of agglutination test, and we found that sometimes there was no agglutination between different serotypes of *Salmonella* and positive sera. Therefore, we used dot blot as similar to the principle of iELISA to detect serum samples, and the coincidence rate was 89%. The difference between the two methods may be due to the stronger specificity of iELISA. The use of the iELISA and commercial ELISA kit to detect the clinical serum samples at the same time demonstrated an agreement of 94.1%, which suggest that the iELISA based on PagN recombinant protein method can perform detection of *Salmonella* infection.

Although the established iELISA method is high in efficiency and sensitivity, it still requires further optimization. For example, the OD readings obtained with non-*Salmonella* sera were close to those of the cut-off value, suggesting a certain level of cross-reactivity. Cross-reactions can be further evaluated by detecting more non-*Salmonella* positive sera and expressing specific PagN epitopes.

## Conclusion

In conclusion, an iELISA-base method using recombinant multiepitope PagN protein as the coating antigen was established successfully. This method has high sensitivity and specificity, and with no serological cross-reaction with other pathogens. We believed that the newly built iELISA method could be a useful detecting tool for large-scale monitoring the epidemiology of *Salmonella* infection in ducks.

## Materials and methods

### Strains, serum samples and antibodies

35 ducks at 7 days of age were selected to confirm *Salmonella*-free ducks by bacterial isolation and identification [35]. After these ducks were raised for 7 days, 5 ducks were randomly selected to collect blood and obtain negative serum control. The remaining ducks were randomly divided into 6 groups (5 per group) according to different pathogenic bacteria, including *Salmonella enteritis*, *Salmonella typhimurium*, *Salmonella Cottbus*, *E. coli*, *Staphylococcus aureus* and *Streptococcus,* above bacteria culture were assigned the 0.2 mL of 6.0 × 10^6^ CFU/mL by three intramuscular injections at 7-day intervals. At week 1 after the last immunization, blood samples from three ducks of each group were used to separate the serum, and then the antibody level of each serum was determined by tube agglutination test [36]. All ducks used for preparing negative serum to *Salmonella* and positive serum to other bacteria were raised in separate isolators, deprived freely of food and water.

The *salmonella* positive serum from ducks in present study is from Harbin Animal Husbandry and Veterinary Research Institute, and the serum was identified by agglutination test.

### Predicting and screening linear B-cell epitopes

Based on the sequence of the *PagN* gene, we predicted and screened linear B-cell epitopes using DNA Star software and IEDB website (http://www.iedb.org/). The antigenic index, surface probability and hydrophilicity values are screened.

### PagN gene amplification and recombinant plasmid construction

Primers were designed to amplify *PagN* gene using NCBI reference sequences of *Salmonella* enteritis (CP050712.1). Upstream primer PagN-F: 5′-CGGGATCCAAAGAAGGGATCTATATCACCGG-3′, downstream primer PagN-R: 5′-CCCAAGCTTAAAGGCGTAAGTAATGCCGAGC-3′, underlined is BamHI and HindIII restriction site, respectively. Using *Salmonella enteritis* (CVCC3377) genomic DNA as a template, the *PagN* fragment was amplified by PCR using PagN specific primers, and the gene fragment was purified using a gel extraction kit. After digestion with BamHI and HindIII, the products were purified and ligated to pET-32a(+) by T4 DNA ligase at 16 °C for 8 h，and then transformed into *E.coil* DH5α competent cells. A single colony on the LB solid medium (containing 100 μg/mL ampicillin) was selected and cultured overnight at 37 °C with shaking at 220 rpm. The positive clones were verified by restriction enzyme digestion and DNA sequencing, yielding pET-32a (+)-PagN.

### Expression and purification of the recombinant PagN protein

The recombinant plasmid pET-32a (+)-PagN was transformed into *E.coli* BL21(DE3) expression strain. Positive clones were picked up and inoculated into 5 mL LB liquid medium with 100 μg/mL of ampicillin overnight at 37 °C. The overnight culture was inoculated into 200 mL LB liquid medium at a ratio of 1:100 and incubated at 37 °C and 220 rpm until the logarithmic growth phase. Expression of recombinant protein was induced by addition of IPTG. The bacterial pellet was collected by centrifugation, the cells were resuspended in phosphate buffered solution (PBS) with 1/10 culture volume and disrupted by sonication. The supernatant and pellet after sonication were collected and subjected to SDS-PAGE to analyze the expression form of the recombinant protein. The recombinant PagN protein was purified using Ni^2+^-NTA agarose gel affinity chromatography. The bound protein was eluted with elution buffer (20 mmol/L Tris-HCL, 200 mmol/L imidazole). Finally, the target protein was renatured in a gradient of 6 M, 4 M, 2 M urea buffer solution at 4 °C and then the purified protein concentration was measured by BCA protein assay kit (Beyotime, Shanghai, China).

### Western blotting assays

The purified recombinant PagN protein was subjected to polyacrylamide gel electrophoresis and then electrotransferred onto a Nitrocellulose (NC) membrane. The NC membrane was blocked at 4 °C in blocking buffer with Tris-buffered saline (TBS) contained 3% BSA and 0.1% Tween 20 (TBST), and then washed 3 times for 5 min in TBST. The blocked membrane was incubated in duck anti-*Salmonella* serum (dilution 1/500, from Harbin Animal Husbandry and Veterinary Research Institute) at 4 °C for 2 h. The membrane was washed 3 times with TBST, and 1:4000 diluted HRP-goat anti-duck IgG (KPL, Gaithersburg, USA) was added and incubated for 2 h at 4 °C. After washed three times, enhanced chemiluminescent (ECL) developer solution was added to develop the color.

### Establishment of the recombinant PagN protein-based iELISA

According to the classical iELISA method [17, 34], the optimal antigen coating concentration and serum dilution of the iELISA test reagents was investigated by checkerboard titration. Briefly, 100 μL of different concentrations (0.25, 0.5, 1.0, and 2.0 μg/mL) of PagN proteins were coated on a 96-well ELISA microplate overnight at 4 °C, 37 °C 1 h or 2 h, respectively. The ELISA plates were then washed three times for 5 min with washing buffer (PBS + 0.5% TWEEN-20). The wells were blocked with 200 μL blocking buffer (1% BSA, 2% BSA, 3% BSA, 3% skimmed milk, 4% skimmed milk and 5% skimmed milk) at 37 °C for 0.5 h, 1 h, 1.5 h, 2.5 h and 3 h, respectively. After three further washes, 100 μL serially diluted the positive and negative serum samples (2-fold dilutions, from 1:25 to 1:400) was added to each well and then incubated at 37 °C for 0.5 h,1 h, 1.5 h or 2 h. After three washes, the samples were incubated with 100 μL of goat anti-duck IgG-horseradish peroxidase conjugate (HRP) (KPL, Gaithersburg, USA) with two-fold serial dilutions (1:2000 to 1:8000) at 37 °C for 0.5 h, 1 h, 1.5 h or 2 h, washed again, and detected with 100 μL 3,3′,5,5′-Tetramethylbenzidine (TMB) color developer at room temperature for 5 min, 10 min,15 min or 20 min and away from light. The reaction was stopped by the addition of 50 μL 2 M H_2_SO_4_. All samples were set up in triplicate and measured with a microplate spectrophotometer (model 680, Bio-Rad) at 450 nm. The corresponding positive value (P) was approximately 1.0, the negative value (N) was less than 0.4, and the maximum difference in optical density (P/N) was not less than 2.1, which was considered to be the best reaction [37, 38].

### Determination of the cut-off value

Under the best condition, the OD_450_ values of 38 negative serum of healthy ducks were determined by the iELISA method, and each serum sample was repeated three times. The mean and standard deviation of the OD_450_ values were calculated. The cut-off value was determined by titration as the mean OD_450_ (^−^x) value plus 3 the standard deviation (SD) of the antibody levels of 38 negative serum samples [34]. If the OD_450_ value of the test sample is higher than the cut-off value, the sample is regards as positive and vice versa.

### Sensitivity analysis and specificity of iELISA test

To accurately assess the diagnostic sensitivity of the assay, the *Salmonella* positive serum was diluted from 1:50 to 1:12800. The highest dilution that produced an OD_450_ value> the cut-off value was considered as the detection limit of the iELISA assay (three replicates each test serum) [34]. In addition, to evaluate the diagnostic specificity, the iELISA method described above was used to simultaneously detect the OD_450_ values of positive serum antibodies against duck-derived *Salmonella Enteritidis, Salmonella Typhimurium*, *Salmonella* Cottbus, *E. coli*, *Staphylococcus aureus* and *Streptococcus* with three replicates each serum sample. The *Salmonella*- positive and -negative serum were set as controls.

### Repeatability analysis

Under the optimal conditions established, three sero-positive serum samples and one sero- negative serum sample were detected by the iELISA. Four repeats were set for each sample, and the average value, standard deviation and coefficient of variation of each sample were calculated.

### Comparison of iELISA with dot-blot

Dot-blot was used to validate the of the developed iELISA. iELISA and Dot-blot were used to detect 100 clinical sera at the same time, and the coincidence rate of them was compared. The steps for Dot-blot are as follows:

1 μg recombinant protein was fully adsorbed on NC membrane at 4 °C. The NC membrane were then washed 4 times for 5 min with TBST buffer. 5% skim milk was added to each well and incubated at 37 °C for 2 h. After 4 further washes, the NC membrane was placed in the positive serum diluted by 5% skimmed milk powder and incubated at 37 °C for 1 h. After 4 washes, the NC membrane was paced in the HRP goat anti-duck IgG by 5% skimmed milk powder and incubated at 37 °C for 1 h. Finally, after washing for 4 times, use enhanced diaminobenzidine (DAB) color developing solution to avoid light and develop 10 min.

### Comparing with commercial ELISA test kits for detection of antibodies against *Salmonella*

To evaluate such iELISA kit using for detection of *Salmonella* in ducks, 613 clinical serum samples were tested by iELISA established in present study and a commercial ELISA kit (catalog number YJ660391;Shanghai Enzyme-linked Biotechnology Co., Ltd., China), which specifically designed to a double-antigen sandwich ELISA for detecting antibodies to *Salmonella* in serum by following the manufacturer’s instructions. A purified protein specific for *Salmonella* was used as a capture antigen and an HRP-conjugate for detecting the antibodies. Agreement between iELISA and the kit results was determined by counting the number of identical results and dividing it by the total number of samples.

### Statistical analysis

Statistical analyses were performed using SPSS software (SPSS18.0). All values given in the text are the mean ± SD from the experiment.

## Supplementary Information

**Additional file 1.**

## Data Availability

All data generated or analyzed during the current study are included in this published article.

## Abbreviations

iELISA: Indirect enzyme-linked immunosorbent assays
PCR: Polymerase chain reaction
qPCR: Quantitative real-time
PCR; LAMP: Loop-mediated isothermal amplification
ICTS: Gold immunochromatographic test strip
PBS: Phosphate buffered solution
TBS: Tris-buffered saline
TBST: Tris-buffered saline Tween-20
ECL: Enhanced chemiluminescent
HRP: Horseradish peroxidase
IPTG: Isopropyl-b-D-thiogalactopyranoside
SDS-PAGE: Sodium dodecyl sulfate polyacrylamide gel elctrophoresis
NC: Nitrocellulose
TMB: 3,3′,5,5′-Tetramethylbenzidine
LPS: Lipopolysaccharide
DAB: Diaminobenzidine
