# Supplementary material for: Establishment of indirect ELISA method for Salmonella antibody detection from ducks based on PagN protein
Source: BMC Vet Res. 2022 Dec 5;18:424. doi: 10.1186/s12917-022-03519-7 (PMC9721058; doi:10.1186/s12917-022-03519-7)
Supplement: Supplementary file 1 — Additional file 1. [file 12917_2022_3519_MOESM1_ESM.pdf]

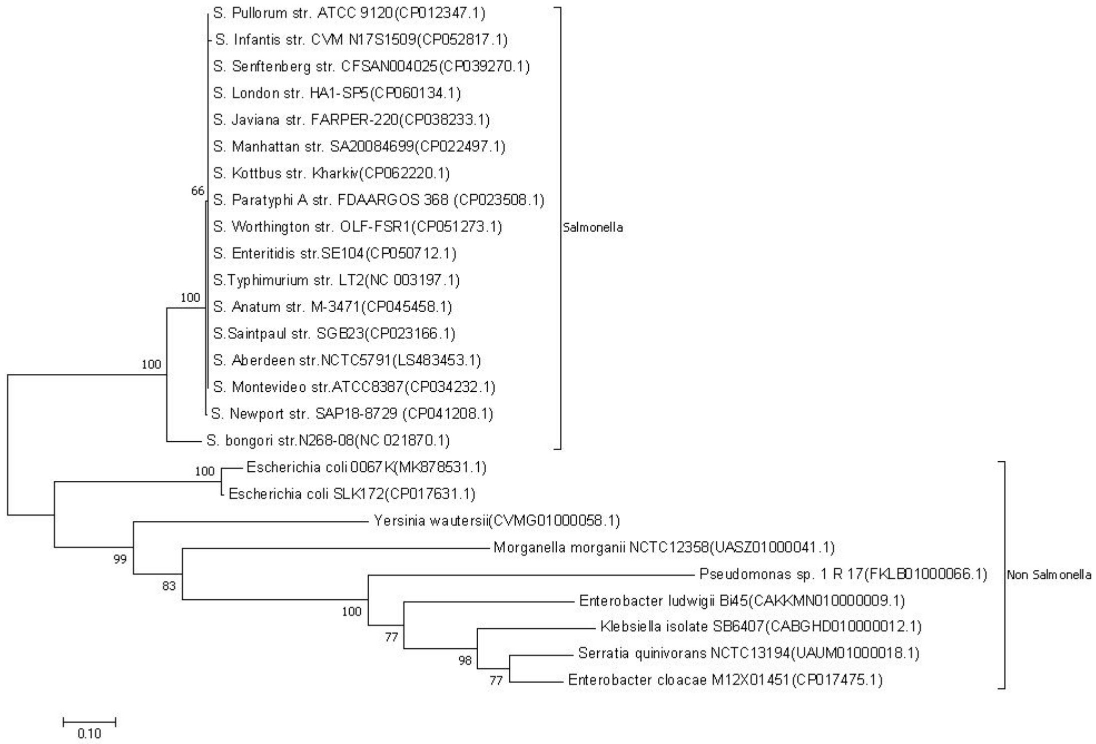

Figure S1. The phylogenetic tree of PagN protein.

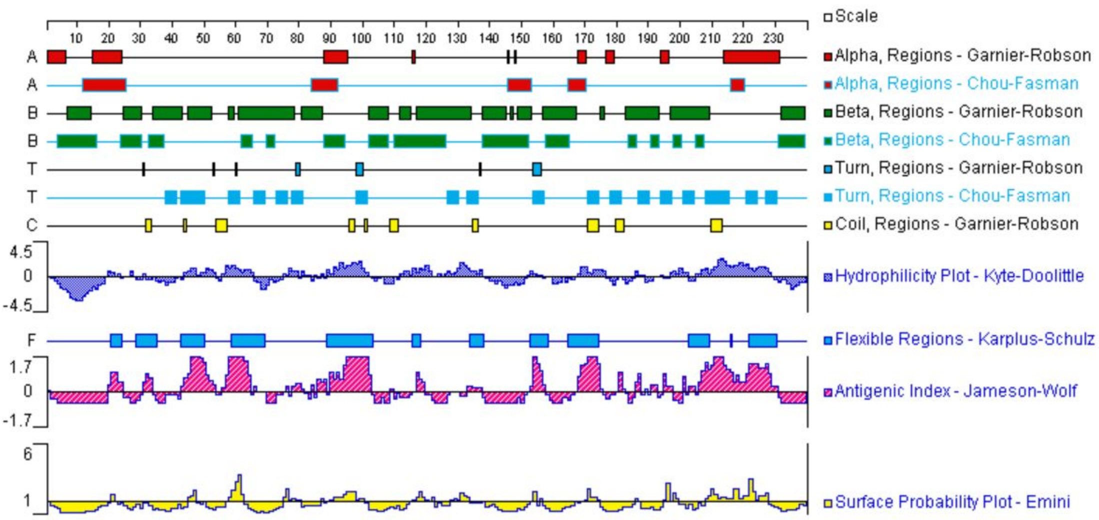

Figure S2. Secondary structure analysis of Pag N protein.

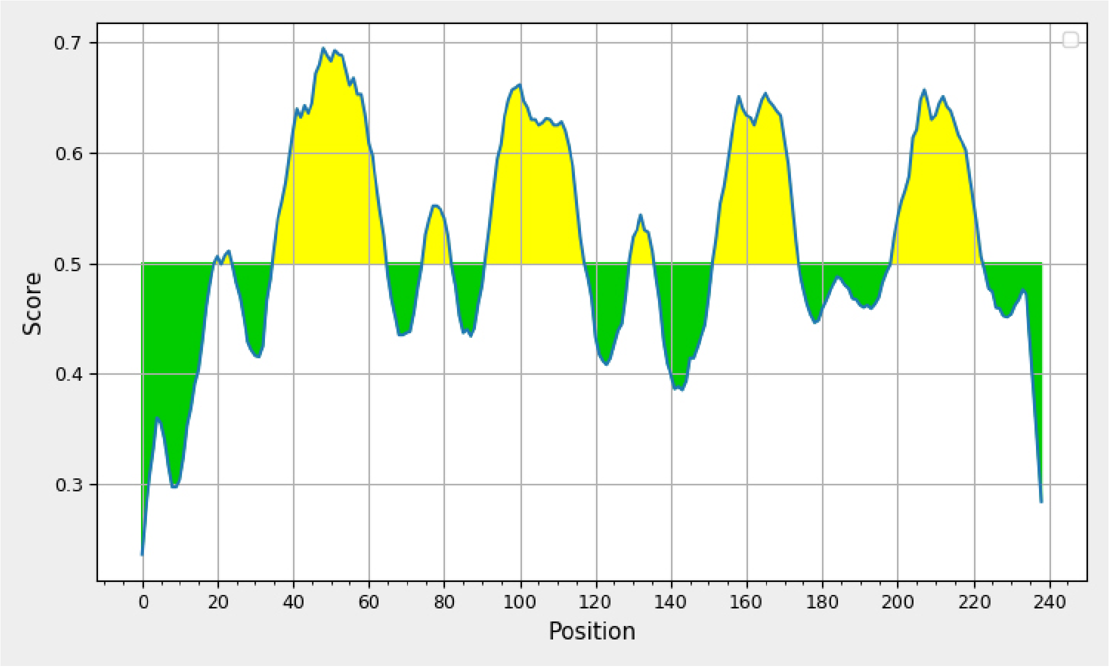

Figure S3. Analysis of B-cell epitopes based on PagN protein.
